# Saving the Breast Saves the Lives of Breast Cancer Patients

**DOI:** 10.1155/2020/8709231

**Published:** 2020-02-27

**Authors:** Mohammad Esmaeil Akbari, Maryam Khayamzadeh, Hamid Reza Mirzaei, Afshin Moradi, Atieh Akbari, Farid Moradian, Neda Khalili

**Affiliations:** Cancer Research Center, Shahid Beheshti University of Medical Sciences, Tehran, Iran

## Abstract

*Introduction*. Surgery has been known as the procedure of choice for breast cancer management since 1700 years before Christ. Nowadays, breast-conserving surgery and mastectomy are performed in selected cases with specific clinical criteria. Here, we compare these two procedures for breast cancer patients with variable features in Cancer Research Center, Tehran, as a single institution experience.

**Methods:**

In this 25-year follow-up retrospective cohort study, we identified breast cancer patients who had undergone breast-conserving therapy or mastectomy. Disease-free survival and overall survival were evaluated using Kaplan–Meier survival analysis and the log-rank test between the two groups. A *p* value less than 0.05 was considered statistically significant.

**Results:**

A total of 3358 breast cancer patients, including 61% breast-conserving therapy and 39% mastectomy cases were identified, with a mean follow-up time of 94 months. The overall survival and disease-free survival of all cases were significantly better in breast-conserved patients, particularly in early-stage breast cancer with favorable clinical, pathological, and biological features. Ten-year disease-free survival and overall survival in breast-conserving therapy and mastectomy cases were 74%, 88% and 58%, 80%, respectively.

**Conclusion:**

Breast-conserving surgery and radiation therapy prove to be an appropriate treatment option for breast cancer patients in terms of overall survival and disease-free survival when indicated.

## 1. Introduction

Breast cancer, the most common malignancy in women worldwide, is a major public health concern. In 2018, 18.1 million new cases were diagnosed with breast cancer, and 9.6 million deaths occurred due to cancer. Also, the 5-year prevalence was estimated to be 43.8 million in the world [1]. Nowadays, management of breast cancer is largely dependent on surgery, radiation, and systemic therapy. Surgery has been introduced as the mainstay of treatment in breast cancer since approximately 1700 BC [2].

Galen, the Greek surgeon (129–210 AD), believed that breast cancer is a systemic disease and proposed that the accumulation of excess black bile accounts for pathogenesis [3]. Based on a very popular document from Avicenna (Canon), surgery has been well defined for an early-stage breast cancer and metastatic to the axilla [4].

All of the physicians, including Hippocratic surgeons (after the 4th century BC), Archigenes of Apamea (1st-2nd century AD), Galen (2nd century AD), Leonides of Alexandria (circa 2nd century AD), and Avicenna (c. 980-June 1037) believed that surgery is effective when the cancer is limited to the breast and not locally advanced; they also recommended to avoid surgery when it is ulcerated or fixed to the axilla and helped the patients in such cases with medicament [3, 4].

Surgery has remained to be the most important procedure in the treatment of breast cancer since the ancient period. In the 1900s, William S Halsted introduced a novel surgical technique, known as radical mastectomy, after conducting a nonrandomized but controlled clinical trial which demonstrated improvement in the locoregional control of the disease. However, no changes were observed in terms of overall survival [5].

After this period, randomized clinical trials of breast-conserving surgery (BCS) were pioneered in Milan by professor Veronesi [6–8] as the National Surgical Adjuvant Breast and Bowel Project (NSABP) chaired by Dr. Bernard Fisher. They defined the benefits and the techniques of this less morbid treatment approach as compared with the more popular radical mastectomy [9].

During the Fisher era, scientists challenged the mastectomy procedure and breast cancer was known as a systemic disease, which is probably controllable by less extensive surgery and by conserving the breast plus radiotherapy. It was confirmed by many trials that BCS in combination with radiation therapy is similar to modified radical mastectomy (MRM) for local/systemic control of breast cancer [6, 7, 9–13]. In all of these trials, not only was there no inferiority for BCS versus mastectomy regarding local recurrences, distant metastases, and overall survival but also many studies showed benefits for breast conservation therapy (BCT) over mastectomy. Besides, some trials demonstrated a slightly more favorable outcome in patients with positive nodes treated with BCS; however, the differences were not statistically significant [14–20].

In a pooled data from the National Cancer Institute (NCI), European Organization for Research and Treatment of Cancer (EORTC), and Institut Gustave Roussy (IGR) trials [15, 21], the odds ratio for overall survival (OS) at 10 years was more favorable in BCT compared with mastectomy. Also, improved survival was observed in BCT patients with positive axillary node over mastectomy cases who did not receive postmastectomy radiation. In another study, although no significant difference in OS was found, local recurrences were less in BCT versus mastectomy (5.9% vs. 6.2%) [15].

Previous studies supported the concept that breast cancer is a systemic disease with local manifestations and radicalistic surgery does not statistically change overall survival; also, these studies confirmed that breast cancer surgery plus radiotherapy (RT) is not an inferior strategy compared with mastectomy.

Recently, some trials have confirmed the superiority of BCS with radiation therapy compared with mastectomy [14, 15, 21].

In this 25-year follow-up study, we aimed to investigate the survival outcomes of female breast cancer patients undergoing BCT or mastectomy.

## 2. Materials and Methods

In this large, retrospective cohort study, we identified breast cancer patients who underwent BCT or mastectomy at the Cancer Research Center between 1991 and 2016. We included patients irrespective of their age, pathologic stage (I-IV), primary tumor size, axillary status, and pathological and biological pattern with no evidence of gross multicentricity or diffuse noninvasive pathology and extensive microcalcification. All male breast cancer patients and those lost to follow-up were excluded from the study.

All of the BCS cases who had positive axillary lymph nodes and tumor size of more than 2 cm received additional chemotherapy (CT) and radiotherapy (RT), approximately 50 Gy with boost dose, after surgery. All the remaining cases who did not receive CT, however, had received RT as an integral part of BCT management.

Mastectomy cases were patients of any age or nodal status with/without neoadjuvant chemotherapy who had extended ductal carcinoma in situ (EDCIS) and also those who preferred mastectomy over BCS. Node-positive mastectomy cases also received CT and RT, whereas node-negative total mastectomy cases with noninvasive breast cancer did not receive CT or RT.

This retrospective cohort study analyzed 3358 breast cancer cases in different stages with 25 years follow-up in two groups of BCS plus RT versus mastectomy cases. For the purpose of analysis, patients were divided into six age groups: <30, 30–39, 40–49, 50–59, 60–69, and ≥70. The number of positive nodes was categorized as follows: 0, 1–3, 4–10, and >10. Data for age at diagnosis, tumor size, pathology (invasive vs. non-invasive), stage of the disease, number of positive lymph nodes, Estrogen Receptor (ER) status, Progesterone Receptor (PR) status, and Her-2 status were extracted for all of the patients. OS was defined as the time from the date of diagnosis to the date of the last follow-up or death (due to any reason). Disease-free survival (DFS) was defined as the time from the date of diagnosis to the date of recurrence. Patient characteristics for the BCT or mastectomy cases were compared using two-sided *t*-tests and chi-square tests. The Kaplan–Meier analysis provided estimates of OS and DFS; statistical significance between BCT and mastectomy groups was accomplished with log-rank testing. All patients with noninvasive disease were excluded from the survival analysis, since noninvasive disease does not affect survival. Data analysis was performed using the SPSS software (version 22), and a *p* value of less than 0.05 was considered as statistically significant in the analysis.

All procedures performed in our study were in accordance with the ethical standards of the institution and/or national research committee and with the 1964 Helsinki Declaration and its later amendments or comparable ethical standards. The ethical committee of Shahid Beheshti University of Medical Sciences approved this study, and written informed consent was obtained from the patients or from parents or legal guardians for minors or incapacitated adults.

## 3. Results

**Figure 1:**
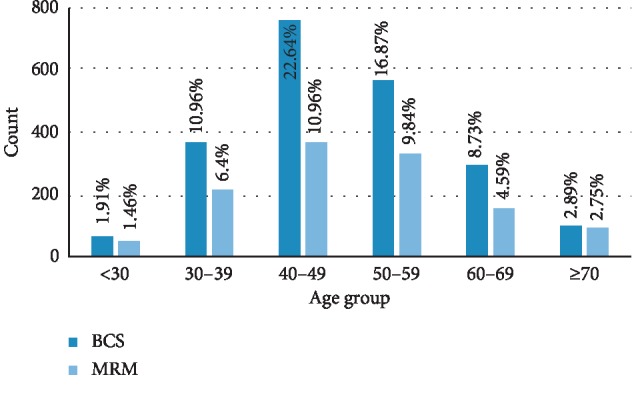
Age distribution of the patients.

**Figure 2:**
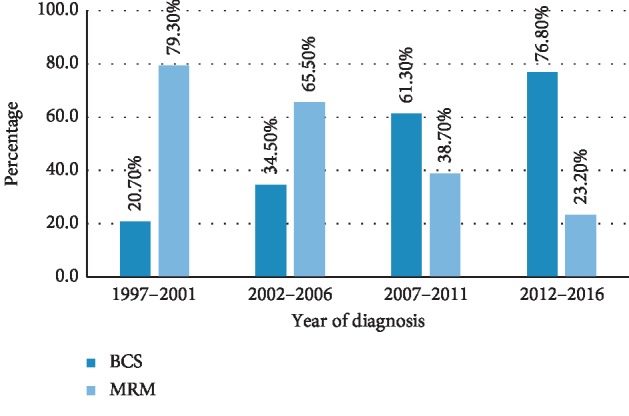
Variation trend based on the kind of surgery.

In this retrospective cohort study, a total of 3358 breast cancer patients who had referred to Cancer Research Center, Tehran, between 1991 and 2016 and had undergone BCT or mastectomy were analyzed. The mean follow-up time for the entire cohort was 94 months. Of the total cases, 2050 (61%) underwent BCT. Only 2% and 5% of patients in BCS and mastectomy groups had stage 4 disease. The majority of patients in the BCT and mastectomy groups had invasive cancer (94% and 96%, respectively). The mean age of patients was 49.12 years (SD = 11.58) with no significant difference between the two groups. Patient and tumor characteristics for both groups are summarized in Table 1. The treatment groups were similar with respect to age. The age distribution of patients is shown in Figure 1. As shown in Figure 2, the trend in the kind of surgery performed for breast cancer patients has changed over the past 20 years, with BCS increasing from 21% to 77%.

**Figure 3:**
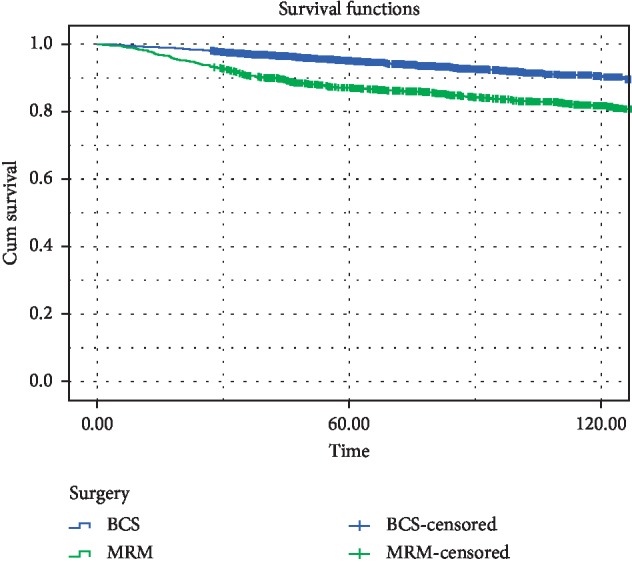
Five- and 10-year overall survival in all patients with BCS and mastectomy.

**Figure 4:**
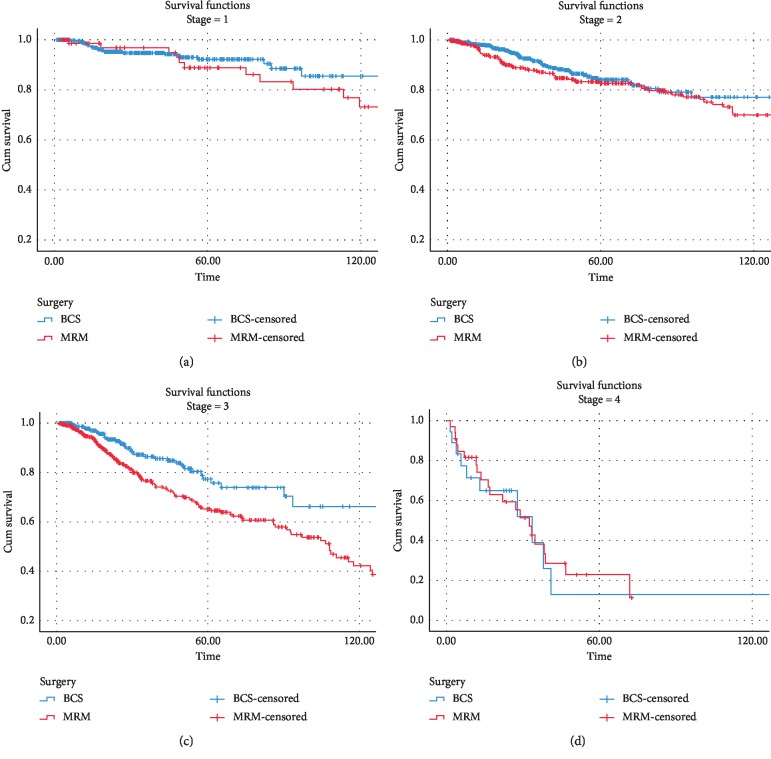
Overall survival based on the kind of surgery and stages. (a) Stage 1 *P*=0.02. (b) Stage 2 *P*=0.012. (c) Stage 3 *P*=0.04. (d) Stage 4 *P*=0.087.

**Figure 5:**
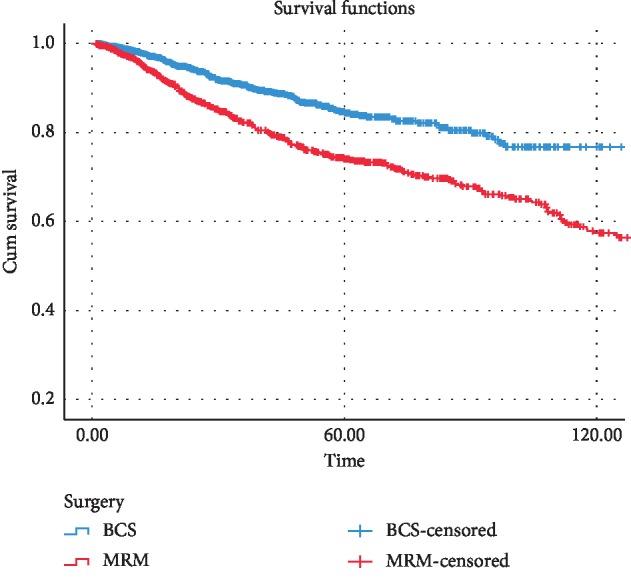
Five- and 10-year disease-free survival in all patients with BCS and mastectomy.

Figure 3 demonstrates the 5- and 10-year overall survival in the two groups, which is significantly better in the BCT group; 5- and 10-year OS rates in BCS and mastectomy cases were 95%, 88%, and 90%, 80%, respectively. When the patients were further subdivided by the pathologic stage, the BCT group continued to show non-inferiority in terms of OS compared with the mastectomy group (Figure 4). In this study, a better DFS was also observed in the BCT group compared with the mastectomy group (Figure 5); regarding DFS, 5- and 10-year rates in BCT and mastectomy cases were 86%, 74%, and 78%, 58%, respectively. 

Table 2 demonstrates the OS and DFS rates in the two groups, BCT and mastectomy, in this study and previous studies.

## 4. Discussion

This is a single institute experience with a mean follow-up of 94 months regarding the kind of surgery for breast cancer patients with different criteria. Historically, the clinical outcomes of BCS followed by radiation therapy and mastectomy are similar to each other, and even the overall survival is similar to that of lumpectomy cases without radiation therapy [10–14].

In line with the previous studies, our study showed that the overall survival was significantly better in BCS compared with mastectomy. After dividing the patients based on their pathologic stage, similar results were observed; meaning that saving the breast saves the life of breast cancer patients with different stages, providing the standard management. Also, the DFS was significantly better in the BCT group as compared with the mastectomy cases, which shows that BCT has a significantly considerable systemic action that could be explained by the abscopal effect. Hence, we highly recommend breast-conserving treatment, particularly in early-stage breast cancer with better biological and pathological status.

In three randomized trials conducted in Italy, 349 mastectomy cases, 1006 cases with quadrantectomy + radiotherapy, 345 lumpectomies + radiotherapy cases, and 273 cases with quadrantectomy without radiotherapy were randomized, and the rate of local recurrences were 2.3%, 3.3%, 12.8%, and 11.7%, respectively. Although the mastectomy and quadrantectomy plus radiotherapy groups had a low incidence of recurrence compared with the other two groups, the statistical significance of the differences was negligible or did not exist at all [13]. In a population-based study in the Netherlands and Denmark, it was shown that BCS plus RT improved the OS in all cases compared with mastectomy, even in T1N0 cases; and also, the 10-year distant metastasis-free survival was significantly better in BCS [22–24].

Based on the current literature, not only clinical criteria such as tumor size and nodal status affects overall survival but also biological and pathological criteria are considered as important factors with a major impact on overall survival. In this regard, some standard managements have been established based on these new findings [25].

One of the most important reasons for improved OS regarding the kind of surgery is theoretically believed to be due to the effect on special aspects such as Quality of Life (QOL), with psychosocial effects on patients who have preserved their breast.

Several previous studies have confirmed psychosocial and spiritual relationship with improvement in QOL and OS during the management of breast cancer patients. According to these research hypotheses, social support and personality traits have an impact on the OS of patients with breast cancer. Social support helps women suffering from breast cancer experience better understanding and coping strategies. In all of these studies, the patients' trust, forgiveness, social support, personality, and other psychosocial and spiritual elements affect QOL and survival in breast cancer patients [26–28].

Studies have shown that patients who preserve their breast have higher self-esteem and disease acceptance compared with patients who have undergone mastectomies [29].

The second important reason for improved OS in BCS is radiation therapy for local control and systemic biological effect on disease control [30, 31].

In a population-based study, the omission of RT after BCS even in the elderly and those who had an increased risk of recurrence was not acceptable because of the increasing potential of local recurrence and breast cancer mortality [32, 33]. In older patients with low-risk breast cancer, RT in addition to hormonal therapy decreased the probability of local recurrences but did not improve OS [34]. Providing the boost dose of irradiation in external beam radiotherapy (EBRT) and intraoperative radiotherapy (IORT) is another effective issue, with its omission increasing the recurrence to 30%, even in cases with less than 3 cm tumor size and a negative margin [35]. In another research, the omission of boost dose increased the recurrence rate from 3.6% to 4.5% compared with cases who received a boost dose [36]. The reduction of mortality in breast cancer patients, who received IORT as boost or radical dose is another valuable effect of RT in breast cancer patients due to the induced immunological effect of local therapy that reduces cancer-related death [37–39].

All of our mastectomy cases who were node-positive and had a tumor size of more than five centimeters received RT; also, all BCS cases either received EBRT (boost and whole breast radiation) or full dose/boost of IORT in selected patients. This supports the evidence that receiving radiation or not could be considered as an independent variable.

The time of radiation therapy after surgery is another challenging issue, which is not approved in many independent studies [40–43]. In a trial with 16 years follow-up, RT confirmed no increased risk of local recurrence when administered 2, 4, and 7 months after surgery and initial systemic therapy [44]. The radiation dose is also important in OS, for example, treatment with less than 8 GY per week is associated with a breast cancer recurrence rate ranging from 26% to 30% [45].

Recently, radiation therapy has been highly considered as a personalized medical treatment because of the molecular markers of each patient and her/his sensitivity to RT; in a study by Kirova, the positive effect of boost was separately confirmed [46].

Many studies have confirmed the immunomodulatory effects of local therapy; radiotherapy promotes the immunological system by inducing immunogenic cell death and subsequently increasing the sensitivity of lymphocytes to tumor cells [37, 47–50]. Studies also have demonstrated that the abscopal effect of local treatment combined with immunotherapy enhances the efficacy of treatment for breast cancer cases in animal models [51].

This study has several limitations. First, regarding its retrospective nature, it is not possible to rule out confounding factors. Moreover, although both groups have received similar adjuvant therapies over time, the changing trend towards BCS in recent years and, thus, the more favorable adjuvant therapies may potentially have had an impact on the overall survival of these patients. Also, we have not investigated the association of biological factors with the survival of patients in each treatment group.

## 5. Conclusion

Our findings are in line with previous studies suggesting an improved survival (OS and DFS) with BCT + RT compared with MRM with/without RT. Thus, BCS should be considered as an appropriate treatment option in breast cancer management when indicated.

## Figures and Tables

**Table 1:** Characteristics of the two groups of patients.

Subject	BCT^¥^	Mastectomy	*P* value
Number (%)	2050 (61)	1308 (39)	
Mean age (year)	48.95	49.47	0.25
Stages			<0.001
I	27.4%	8.7%	
II	49.1%	40.8%	
III	21.6%	45.7%	
IV	1.8%	4.8%	
Tumor size			<0.001
T1	39.6%	18.2%	
T2	55.3%	59.2%	
T3	5.1%	22.3%	
Number of positive nodes			<0.001
0	55.5%	34.8%	
1–3	26.2%	26.7%	
4–10	13.6%	26%	
>10	4.8%	12.8%	
Pathology			<0.002
Noninvasive	6.1%	3.7%	
Invasive	93.9%	96.3%	
Radiation therapy	98.9%	88.3%	<0.001
ER+	74.6%	66%	<0.001
PR+	69.1%	56.5%	<0.001
Her-2+	19.9%	30.9%	<0.001
Post NACT^ƚ^	8.8%	13.8%	<0.001
Chemotherapy	93.4%	97.9%	<0.001

^¥^Breast conserving therapy. ^Ł^Postneoadjuvant chemotherapy.

**Table 2:** Results of 5-year OS and DFS in prospective randomized trials comparing breast conservative surgery and radiotherapy (BCT) with mastectomy.

Trial	Endpoint (years)	Overall Survival (%)		Disease-free Survival (%)	
		BCS & RT	Mastectomy	BCS and RT	Mastectomy
NCI milan [15]	18	65	65		N/A
Institut Gustav Roussy [15]	15	73	65		N/A
NSABP B-06 [15]	12	63	59	50	49
NCI USA [15]	10	77	75	72	69
EORTC [15]	8	54	61		N/A
Danish breast cancer group [15]	6	79	82	70	66
Akbari et al. [14]	13	86	78		N/A
Akbari et al. (Current study)	25	95	88	86	76

OS: Overall survival, DFS: disease-free survival, N/A: not applicable, BCS and RT: breast-conserving surgery and radiotherapy.

## Data Availability

The data used to support the findings of this study are available from the corresponding author upon request.
